# Nanoemulsion of *Lavandula angustifolia* Essential Oil/Gold Nanoparticles: Antibacterial Effect against Multidrug-Resistant Wound-Causing Bacteria

**DOI:** 10.3390/molecules28196988

**Published:** 2023-10-09

**Authors:** Balqis A. Fadel, Bassma H. Elwakil, Esraa E. Fawzy, Marwa M. Shaaban, Zakia A. Olama

**Affiliations:** 1 Department of Botany & Microbiology, Faculty of Science, Alexandria University, Alexandria 21568, Egyptesraa.esmat@alexu.edu.eg (E.E.F.);; 2 Department of Medical Laboratory Technology, Faculty of Applied Health Sciences Technology, Pharos University in Alexandria, Alexandria 21526, Egypt; 3 Department of Pharmaceutical Chemistry, Faculty of Pharmacy, Alexandria University, Alexandria 21521, Egypt; marwa.mamdouh@alexu.edu.eg

**Keywords:** nano-gold/nano-*Lavandula angustifolia* formula, antibacterial, antibiofilm, wound healing

## Abstract

Hospitalized patients are severely impacted by delayed wound healing. Recently, there has been a growing focus on enhancing wound healing using suitable dressings. *Lavandula angustifolia* essential oil (LEO) showed potential antibacterial, anti-inflammatory, and wound healing properties. However, the prepared gold nanoparticles possessed multifunctional properties. Consequently, the present investigation aimed to synthesize a novel nanosystem consisting of nano-*Lavandula angustifolia* essential oil and gold nanoparticles prepared through ultrasonic nanoemulsifying techniques in order to promote wound healing and combat bacterial infection. LEO showed potent antibacterial activity against *Klebsiella pneumoniae*, MRSA and *Staphylococcus aureus* with minimum inhibitory concentration (MIC) values of 32, 16 and 16 µg/mL, respectively, while exhibiting low activity against *Proteus mirabilis*. Interestingly, the newly formulated nano-gold/nano-*Lavandula angustifolia* penetrated the preformed *P. mirabilis* biofilm with a full eradication of the microbial cells, with MIC and MBEC (minimal biofilm eradication concentration) values reaching 8 and 16 µg/mL, respectively. The cytotoxic effect of the novel nanoformula was also assessed against WI-38 fibroblasts vero (normal) cells (IC_50_ = 0.089 mg/mL) while nano-gold and nano-*Lavandula angustifolia* showed higher results (IC_50_ = 0.529, and 0.209 mg/mL, respectively). Nano-gold/nano-*Lavandula angustifolia* formula possessed a powerful wound healing efficacy with a 96.78% wound closure. These findings revealed that nano-gold/nano-*Lavandula angustifolia* nanoemulsion can inhibit bacterial growth and accelerate the wound healing rate.

## 1. Introduction

The ability of the human body to protect itself from the external environment is compromised when the epithelium and connective tissues are damaged. The incidence of an infection, particularly from chronic wounds, is the prevailing and unavoidable obstacle to the wound healing process [1]. Bacteria are frequently present in both the normal skin microbiota and in wounds. However, the significant accumulation of bacteria with the subsequent development of a biofilm might hinder the process of wound healing [2]. Despite recent advancements in wound treatment, bacterial and fungal infections are still recognized as highly prevalent and distressing conditions that have a considerable impact on mortality and morbidity rates [3]. There is a high need for effective and tailored wound treatments due to the unique biological characteristics of non-sterile wound environments and the complex nature of the wound healing process for both chronic and acute wound infections [4]. Noble metal nanoparticles (NPs), such as gold (Au), are among the most significant well-known infected-wound treatments.

Gold nanoparticles (AuNPs) have received a lot of interest due to their versatile biological activity. Because of their superior properties in treatments, detection as well as imaging, gold nanoparticles have been employed in several applications such as material sciences, physics, medical and biological sciences [5].

On the other hand, essential oils (EOs) are a class of low-molecular-weight secondary metabolites derived from plants, encompassing compounds such as monoterpenes and sesquiterpenes. Due to their sensory attributes, including flavor, aroma and antimicrobial properties, these substances have been involved in many fields such as agriculture [6], medicine [7], wound healing [8], cosmetics [9], pharmaceuticals, tissue engineering for the development of skin scaffolds [10,11] and the food packaging industry [12]. Lavender essential oil (LEO) is derived from the blossoms of *Lavandula angustifolia* and is recognized for its potential to alleviate anxiety, provide pain relief, exhibit antioxidant properties and demonstrate anticancer activity [13,14]. Tragically, the utilization of lavender essential oils in pharmaceutical applications is significantly constrained due to their suboptimal physicochemical characteristics, such as their limited solubility in water. Consequently, the development of an appropriate formulation is necessary to address the inadequate biopharmaceutical properties of these oils and fully capitalize on their therapeutic capabilities [15]. Aqueous nanoemulsions have been a topic of growing attention in the field of formulation techniques due to their advantageous properties, such as simple fabrication and handling, as well as relatively low production costs. These characteristics make them a promising option for the efficient delivery of essential oils. Ultrafine isotropic dispersed systems are characterized by the presence of two non-miscible liquids. Typically, these systems consist of an oily phase dispersed within an aqueous phase, forming nanometer-sized droplets [16]. The stability of these droplets is maintained by a surfactant-based interfacial coating. The majority of the physical and pharmacological characteristics of nanoemulsions may be attributed to the diminutive dimensions of the scattered globules. The colloidal dispersion’s significant specific surface area facilitates the solubility and penetration of active chemicals supplied, leading to enhanced bioavailability and pharmacological effectiveness [16]. The kinetic stability of this colloidal dispersion in comparison to traditional emulsion systems may be attributed to the nanoscale size of the dispersed globules. Nanoemulsions are able to prevent coalescence for extended periods of storage due to the Brownian movements of the nanometric droplets, which counteract gravitational separation forces [17]. In this context, Hajiali and colleagues [18] have developed a formulation of lavender essential oil (LEO) in alginate (SA)/PEO nanofiber to assess the antibacterial and anti-inflammatory properties of LEO, as well as its potential to promote the healing of burn wounds. Interestingly, the findings of this study demonstrated that the nanofibers had inhibitory effects on *Staphylococcus aureus* and effectively regulated cytokine levels with no observed erythema on the wounded skin [18].

Hence, the present study aimed to evaluate the antimicrobial and wound healing efficacy of the newly synthesized *Lavandula angustifolia* essential oil nanoemulsion, combined with gold nanoparticles.

## 2. Results and Discussions

### 2.1. Antibacterial Activity of Lavandula angustifolia Essential Oil

*Lavandula angustifolia* essential oil showed the highest antibacterial activity against *K. pneumoniae,* MRSA and *S. aureus* with inhibition zone (IZ) diameters of 20, 25 and 28 mm, respectively, while MIC values were recorded as 32, 16 and 16 µg/mL, respectively (Table 1). On the other hand, *P. mirabilis* was the most resistant strain; hence, it was chosen for further analyses.

Jianu et al. [19] mentioned that the Lavandula angustifolia essential oil showed antimicrobial activity against *Shigella flexneri*, *Staphylococcus aureus*, *E. coli* and *Salmonella typhimurium*, with significant bactericidal effects, while *Streptococcus pyogenes* was resistant. On the other hand, Ciocarlan et al. [20] revealed that *L. angustifolia* essential oil reported high antimicrobial activity against *Aspergillus niger*, *Alternaria alternata*, *Penicillium chrysogenum*, *Bacillus* sp. and *Pseudomonas aeroginosa* strains at 0.75–6.0 μg/mL, 0.08–0.125 μg/mL and 0.05–4.0 μg/mL, respectively.

### 2.2. Chemical Analysis of Lavandula angustifolia

**Figure 1:**
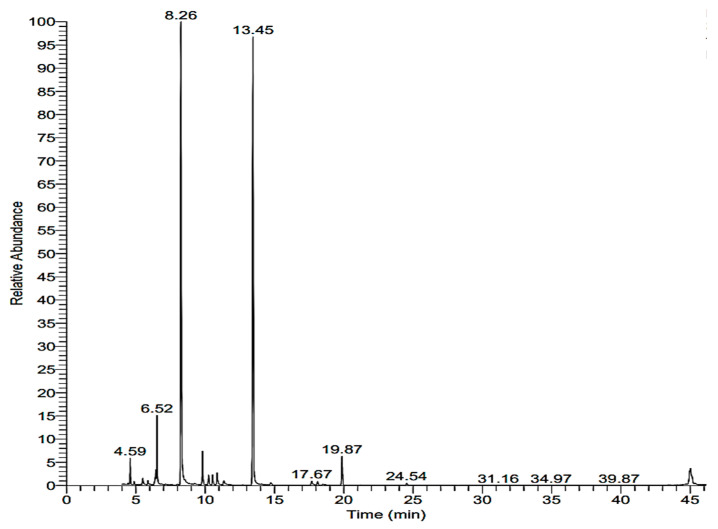
GC–MS chromatogram of *Lavandula angustifolia* essential oil.

Data in Figure 1 show that cyclohexanol, 2-methyl-5-(1-methylethenyl) (carvomenthol), 1,6-octadien-3-ol, 3,7-dimethyl (linalool) and 1,8-cineol (eucalyptol) had been identified by referring to the corresponding acquisition time with area percentages of 11.4, 60.2 and 38.5, respectively (Table 2). Jianu et al. [19] reported that the essential oil of *L. angustifolia* included caryophyllene (24.1%), beta-phellandrene (16%) and eucalyptol (15.6%) as its primary constituents. In a study conducted by Ciocarlan et al. [20], it was revealed that the most important constituents of *L. angustifolia* EO were monoterpenes (84.08–92.55%), followed by sesquiterpenes (3.30–13.45%) and some aliphatic compounds (1.42–3.90%). Moreover, they reported that the main constituents of *L. angustifolia* essential oil were linalool, linalyl acetate, 1,8-cineole, borneol, camphor, lavandulyl acetate, *β*-caryophyllene, *β*-ocimene, *α*-fenchone, terpinen-4-ol, caryophyllene oxide, limonene, pinenes, geranyl acetate, *β*-farnesene, santalene, lavandulol, camphene, geraniol and *α*-terpineol. The content of oxygenated monoterpenes prevails in *L. angustifolia* essential oil and varies between 36.33 and 92.90% [21]. In another study, 1,4-cyclohexadiene, 1-methyl-4-(1-methylethyl)- was detected in essential oil extracted from the aerial parts of lavender at their full flowering stage [22].

### 2.3. Synthesis and Characterization of the Prepared Nanosystems

**Figure 2:**
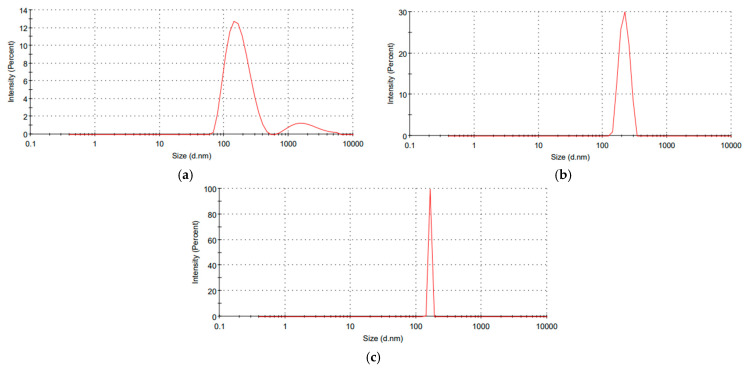
Zeta size distribution of *Lavandula angustifolia* nanoemulsion (**a**), gold nanoparticles (**b**) and nano-gold/nano-*Lavandula angustifolia* (**c**).

**Figure 3:**
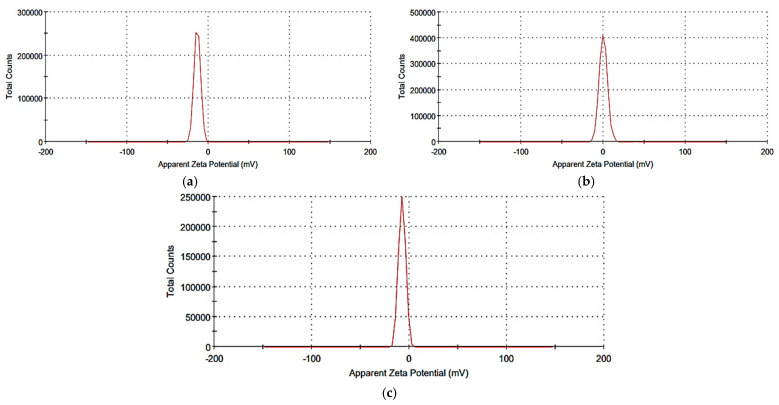
Zeta potential of *Lavandula angustifolia* nanoemulsion (**a**), gold nanoparticles (**b**) and nano-gold/nano-*Lavandula angustifolia* (**c**).

All the synthesized nanosystems were characterized through Zetasizer, Fourier transform infrared spectroscopy (FTIR) and transmission electron microscopy (TEM) analyses. Light scattering (DLS) of the synthesized nanoparticles revealed that the nanoparticles’ size, polydispersity index and zeta potential of nano-*Lavandula angustifolia* were 199.5 nm, 0.404 and −13.6 mV; of nano-gold were 164.8 nm, 0.25 and +0.088 mV; and of nano-gold/nano-*Lavandula angustifolia* were 176.4 nm, 0.429 and −7.38 mV, respectively (Figure 2), while TEM micrographs revealed that the average diameter ranges of nano-*Lavandula angustifolia*, nano-gold and nano-*Lavandula angustifolia*/nano-gold were 96.8, 19.2 and 12.7 nm, respectively (Figure 3).

The presence of a spherical morphology in the synthesized nanoparticles suggests that they possess a low surface energy and exhibit significant thermodynamic stability. This observation further supports the notion that the synthesized gold nanoparticles have a substantial zeta potential. Bhat et al. [23] provided evidence that the extract derived from the edible fungus *P. florida* has the potential to be used in the biosynthesis of spherical gold nanoparticles (AuNPs), with 10 to 50 nm particle size. In another study, Rastogi and Arunachalam [24] used an aqueous extract of garlic (*Allium sativum*) for the manufacture of gold nanoparticles (AuNPs) by the application of microwave irradiation. The nanoparticles that were generated had an average diameter of 23.2 nm and exhibited mostly spherical morphology.

The findings of Chong et al.’s study [25] indicated that the use of mixed surfactants resulted in a superior performance in terms of the storage stability of nanoemulsions compared to pure surfactants with a hydrophilic–lipophilic balance (HLB) value of 15, which was in agreement with previous studies [26,27]. Previous research has shown that the synergistic effects of mixed surfactants may be influenced by variations in headgroup sizes [28]. The presence of greater disparities in headgroup sizes had a significant role in the amplification of synergistic effects. This may be attributed to the favorable interfacial packing between small molecule surfactants and larger surfactants at the interface separating the oil and water phases. The observed significant difference in headgroup size between Tween 80 and Span 80 contributes to the synergistic action of these two compounds, resulting in an improved stability of nanoemulsion systems [29]. Additionally, the stability of nanoemulsion was improved by the use of mixed surfactants, which strengthened the interfacial coating of the nanoemulsion [30]. This phenomenon might be attributed to the inherent hydrophilic and lipophilic properties of mixed surfactants, which promote the adsorption between the oil and water phases. Mixed surfactants exhibit enhanced dispersity and solubility in the continuous phase, facilitating effective mixing with both water and oil [27,31].

Another feature that was assessed in the study was surface charge density, which was determined by the measurement of zeta potential. This parameter helps to identify the presence of repulsive forces that counteract instability events. As an example, the acquired data for the zeta potential (expressed in absolute values) exhibited values around 11. The acquisition of a negative charge may be attributed to the presence of Tween 80 and Span 80 molecules and/or the formation of hydrogen bonds between their oxyethylene groups and hydroxyl ions [32]. Furthermore, it was previously reported that the magnitude of the surface charge density was subject to variations based on the specific kind of oily phase. Marinova et al. [33] observed that the zeta potential is significantly affected by the pH of the aqueous phase. This suggests that the presence of hydroxyl ions at the oil/water interface leads to a negative surface charge, which could explain the relatively higher absolute value of zeta potential observed in the nano-*Lavandula angustifolia*. This is due to the fact that pH has a substantial impact on the electrical charge of the constituent groups within these systems [34].

**Figure 4:**
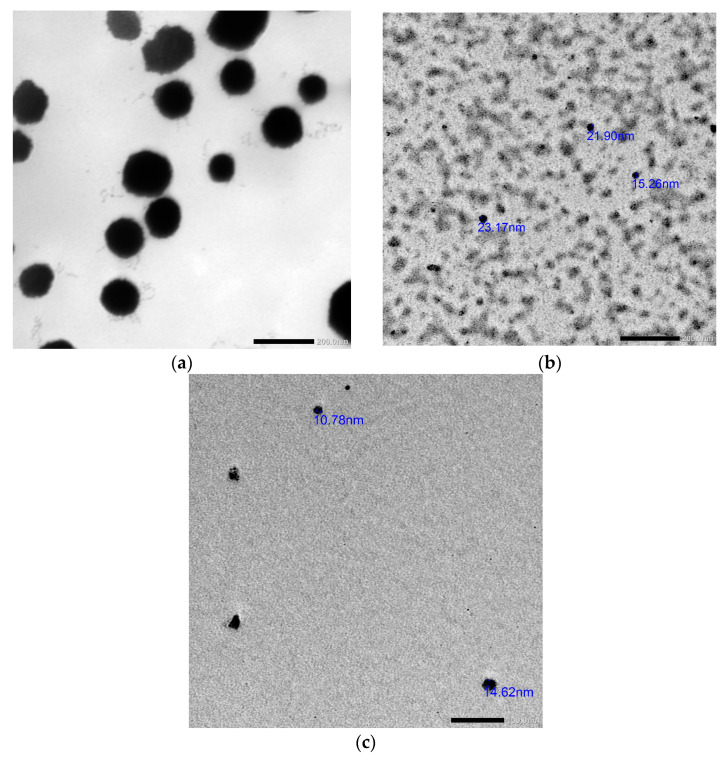
TEM images of *Lavandula angustifolia* nanoemulsion (**a**), gold nanoparticles (**b**) and nano-gold/nano-*Lavandula angustifolia* (**c**).

**Figure 5:**
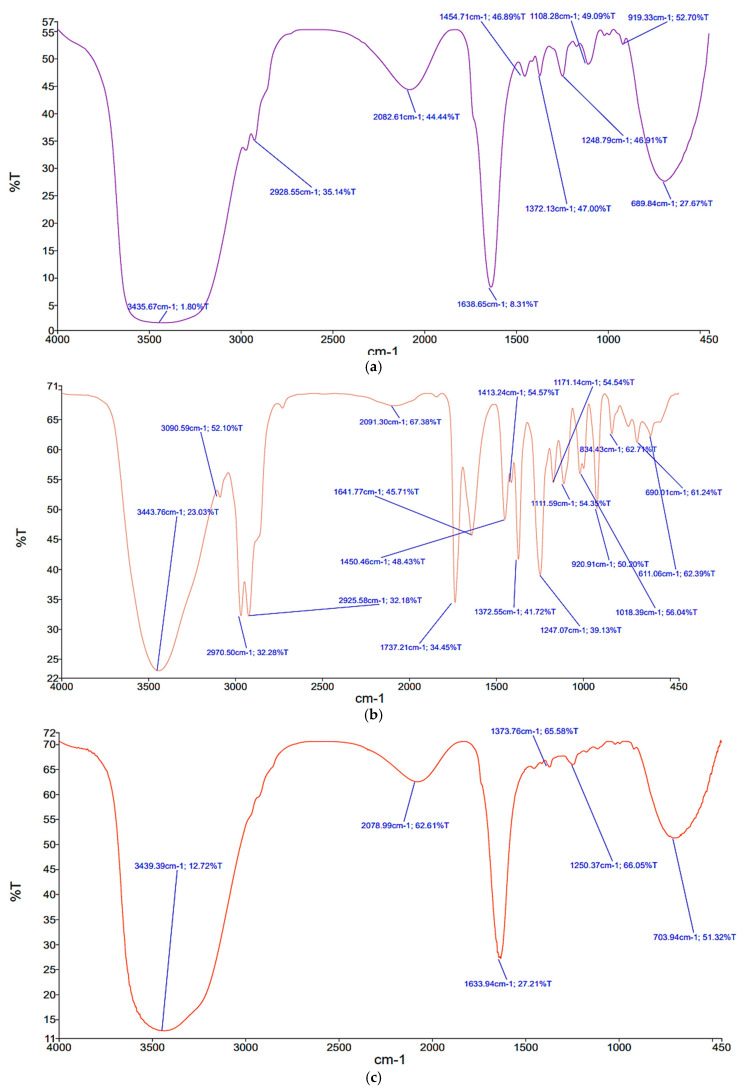
FTIR spectroscope graphs of *Lavandula angustifolia* nanoemulsion (**a**), gold nanoparticles (**b**) and nano-gold/nano-*Lavandula angustifolia* (**c**).

FTIR spectral analysis of the nanoemulsion (Figure 4a) revealed a characteristic broad band from 3200 to 3600 cm^−1^, corresponding to O–H stretching in addition to the common C=O stretching band of around 1700 cm^−1^. These absorption bands could be assigned to the functional groups of Span 80 and Tween 80 surfactants attached to the nanoparticles [35]. The FTIR (Figure 5b) showed the functional groups responsible for gold nanoparticles synthesis, stabilizing and capping using LEO. It demonstrated the common absorption bands at 3423, 2871, 1636 and 796 cm^−1^. These observed bands could be attributed to O–H stretching (3423 cm^−1^), CH stretching (2871 cm^−1^), C=C stretching (1636 cm^−1^) and vibrations of the Au–O bond (1057 cm^−1^), which agrees with the literature values [36]. FTIR indicated the importance of *Lavandula angustifolia* essential oil ingredients in stabilizing and capping the produced nano-*Lavandula angustifolia*/nano-gold within the matrix (Figure 5c). Cossetin et al. [37] reported that *L. dentata* (5%) essential oil-loaded nanoemulsion (NE-LO) had a droplet size of 64.99 nm and a polydispersity index (PDI) of 0.26. The formulation of NE-LO enabled a good protection for α-pinene, sabinene 1.8-cineole, fenchone and camphor at room temperature [38]. The primary components of *Lavandula dentata* essential oil were found to be 1.8 cineol (50–52%), fenchone (17–16%) and camphor (15–13%), while in the present study, the chemical composition of the tested essential oil differed from the one reported by Cossetin et al. [37], which may be attributed to the observed differences. The reason behind such an observation could be the increased lipophilicity with the increase in the length of the carbon chain, which would require a greater energy or S_mix_ concentration to lower the interfacial tension and generate a stable nanoemulsion system [39].

### 2.4. Antibacterial and Antibiofilm Activity of the Synthesized Nanosystems

**Figure 6:**
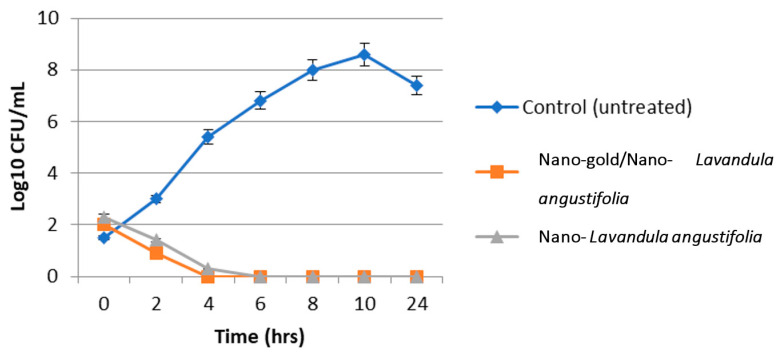
Microbial lethality curve against *P. mirabilis*.

Data in Figure 5 show that *P. mirabilis* bacterial growth reached 0 after 4 and 6 h incubation with nano-gold/nano-*Lavandula angustifolia* and *Lavandula angustifolia* nanoemulsion, respectively. *P. mirabilis* culture grown without nano-gold/nano-*Lavandula angustifolia* exhibited normal morphology, and the microbial cells were arranged while under the presence of nano-gold/nano-*Lavandula angustifolia* (16 µg/mL); *P. mirabilis* showed dramatically restricted bacterial growth inhibition (Figure 6). The observed outcomes may be explained by the inherent capacity of AuNPs to induce cell death via penetration of the bacterial cell wall. Indeed, gold nanoparticles (AuNPs) have the ability to engage with the negatively charged groups present in the constituents of the cell wall, resulting in alterations to the structural composition of the cell surface. A significant quantity of gold nanoparticles (AuNPs) has the ability to form robust bonds with polycyclic aromatic hydrocarbons present on the bacterial cell wall, resulting in the formation of polyelectrolyte complexes. These complexes have the potential to impede the transportation of vital solutes into the cell, as shown by previous studies [40,41,42].

It was proven that nano-gold/nano-*Lavandula angustifolia* inhibited the bacterial biofilm (Table 3). Biofilm formation plays an essential role in invading the host immune defenses and in increasing the antibiotic resistance, which helps in the persistence of microbial infections [43]. Physicochemical features of nanoparticles, including surface charge, PEGylation (the surface modification of particles with polyethylene glycol to enhance stability) and hydrophobicity, also have a role in influencing the nonspecific absorption and probable destruction of particles in macrophage cells. Consequently, the present approach of combining two nanosystems would synergistically combine the benefits of both active and passive targeting, thereby facilitating the specific targeting of cells within the body [44]. Hence, inhibiting the bacterial biofilm by using the newly prepared nano-gold/nano-*Lavandula angustifolia* may pave the way to more reliable and effective methods against persistent and virulent microbial infections.

### 2.5. Cytotoxic Effect of Nano-Gold/Nano-Lavandula angustifolia

**Figure 7:**
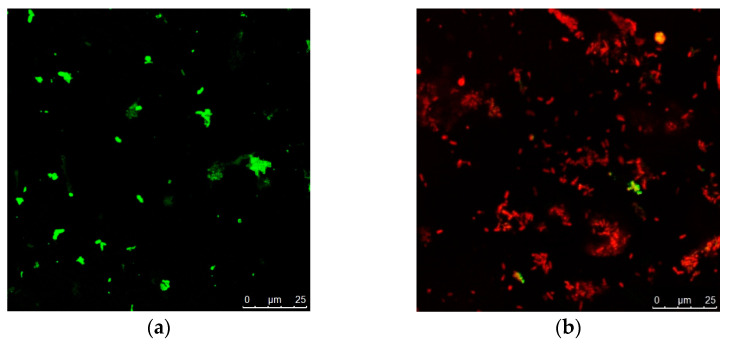
Confocal microscopic examination of *P. mirabilis* ((**a**) control untreated cells, (**b**) nano-gold/nano-*Lavandula angustifolia*-treated cells).

The IC_50_ values of the prepared nanosystems were 0.209, 0.529 and 0.089 mg/mL for nano-*Lavandula angustifolia*, nano-gold, nano-*Lavandula angustifolia*/nano-gold, respectively (Figure 7). Chianese et al. [22] tested the cytotoxic effect of *L. angustifolia* EO and reported no observed cytotoxic effects after 2 and 24 h incubation. Ovidi et al. [16] reported that lavandin EO nanoemulsions showed asserted cytotoxic effects on human lymphoblastic leukemia (CCRF-CEM), human neuroblastoma (SHSY5Y), breast cancer (MCF7) and human colorectal adenocarcinoma (Caco-2) cell lines. EC_50_ values showed dose-dependent and not time-dependent effects, ranging from 1.30 × 10^−2^ ± 0.09 × 10^−2^% after 24 h, to 1.25 × 10^−2^ ± 0.08 × 10^−2^% after 72 h in CCRF-CEM; from 1.42 × 10^−2^ ± 0.08 × 10^−2^% after 24 h to 1.97 × 10^−2^ ± 0.10 × 10^−2^% after 72 h in SHSY5Y cells; and from 13.52 × 10^−2^ ± 1.97 × 10^−2^% after 24 h to 12.45 × 10^−2^ ± 0.99 × 10^−2^% after 72 h for Caco-2 cells. In contrast, the two breast cells lines have shown a slight time-dependent effect, ranging from 3.62 × 10^−2^ ± 0.76 × 10^−2^% after 24 h to 4.53 × 10^−2^ ± 0.68 × 10^−2^% after 72 h of treatment for the breast cancer cell line (MCF7) and from 6.73 × 10^−2^ ± 1.04 × 10^−2^% after 24 h to 3.47 × 10^−2^ ± 0.06 × 10^−2^% after 72 h of treatment for the normal breast epithelial cell (MCF10A). In a study of *Enterococcus*-derived gold nanoparticles (AuNPs), the findings indicated that the application of AuNPs resulted in the stimulation of reactive oxygen species (ROS) and caspase-3 expressions, while concurrently diminishing the mitochondrial membrane potential. The observation of morphology changes associated with apoptosis after treatment with AuNPs in HT-29 cells suggested the potential use of AuNPs as a pro-apoptotic drug in the treatment of colon cancer [45]. Vilela et al. [46] reported that there was no statistically significant difference seen in the vitality of adipose tissue-derived stem cells (ASCs) and myometrial cells after treatment with lavender oil (LO). Moreover, the application of lavender oil and its derivative, niosomal lavender oil (LON), demonstrated cell viability comparable to that of the control culture, indicating their safety for use in cell culture. The only anomaly seen was a viability decrease in myometrial cells, specifically in the case of 0.063% LO. Despite a notable drop in viability at the highest dosage, it remained over 70% in both LO and LON treatments.

### 2.6. In Vitro Wound Healing Activity of Nano-Gold/Nano-Lavandula angustifolia

**Figure 8:**
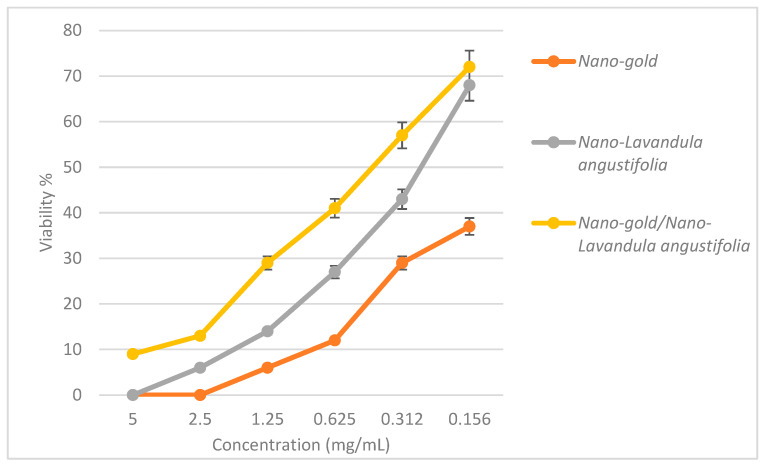
Cytotoxic effect of the prepared nanosystems.

**Figure 9:**
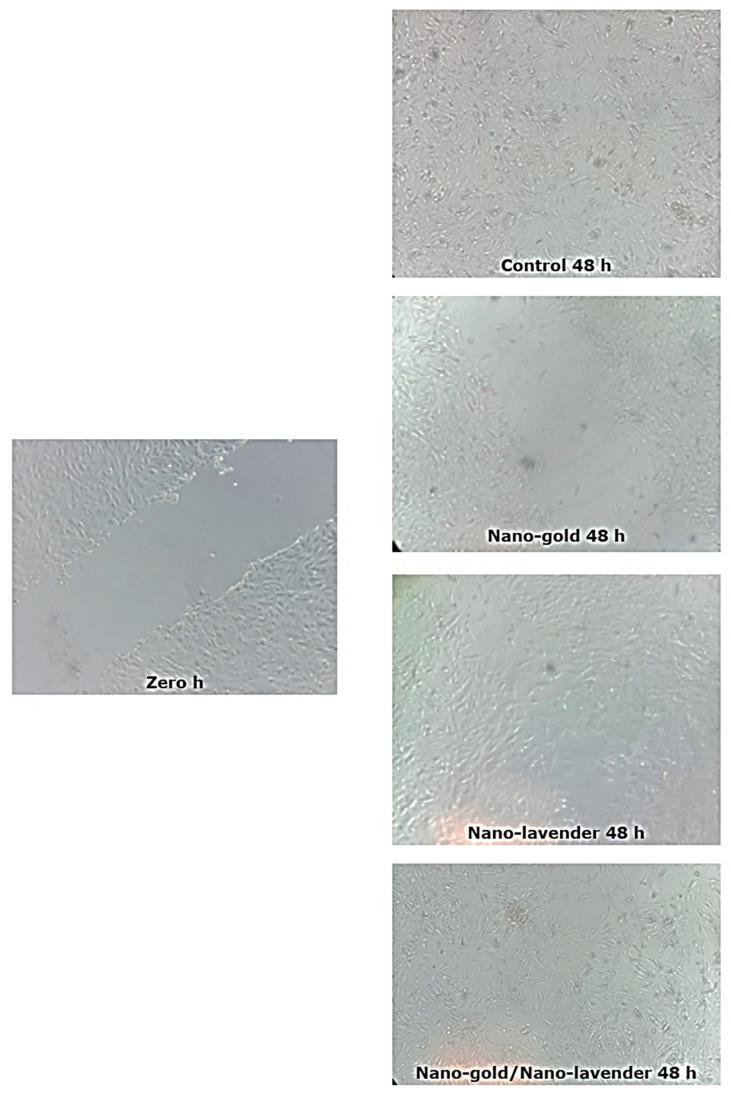
Wound closure effect of the prepared nanosystems.

The newly synthesized nano-gold/nano-*Lavandula angustifolia* showed a powerful wound healing efficacy with a 96.78% wound closing relevant to the control (non-treated wounds) with 70.74% wound closure (Table 4, Figure 8 and Figure 9). It is worth mentioning that *Lavandula angustifolia* oil accelerates the healing rate and can increase the collagen expression, which preserves the skin elasticity and improves the activity of rebuilding tissue proteins [47].

## 3. Materials and Methods

### 3.1. Chemicals

Lavender oil (*Lavandula angustifolia*) (CAS number 8000-28-0), chloroauric acid (HAuCl_4_), polyethylene glycol sorbitan monooleate (Tween 80) and sorbitan monooleate (Span 80) were purchased from Sigma-Aldrich (St. Louis, MO, USA).

### 3.2. Microorganisms

Five bacterial strains, namely *P. mirabilis*, *K. pneumoniae*, MRSA, *E. coli*, *S. aureus* and *A. baumannii* (Table S1), were isolated, identified using Vitek 2 automated system (bioMerieux, Marcy l’Etoile, France) and were kindly provided by Microbiology Routine Laboratory, Main University Hospital, Alexandria University, Egypt. All the tested bacterial strains were maintained on brain–heart infusion glycerol broth with monthly transfer in fresh media.

### 3.3. Antibacterial Activity of the Tested Essential Oil (EO)

Bacterial sensitivity was assessed using the agar disc diffusion technique, which is extensively used to assess antibacterial activity. Petri dishes containing Muller Hinton agar (MHA) were inoculated with 0.1 mL freshly prepared bacterial suspension (0.5 McFarland). Sterile discs (6 mm) saturated with 15 μL *Lavandula angustifolia* (EO) were placed on the surface of the inoculated MHA plates. The inhibition zone diameters were measured in mm after incubation at 37 °C for 24 h. All the obtained results are the mean of three trials [48]. Furthermore, minimal inhibitory concentration (MIC) and minimal bactericidal concentration (MBC) assessments were performed in a 96-well microplate assay. A total volume of 100 μL of the tested EOs solution were serially diluted, and 50 μL of bacterial suspension was inoculated into each well. Finally, the microplate was incubated for 24 h at 37 °C; after the incubation, the first dilutions showed that no bacterial growth were reported as MICs [49].

### 3.4. Chemical Characterization of Lavandula angustifolia Essential Oil

The chemical composition of *Lavandula angustifolia* EO was investigated using gas chromatography TQ8040 NX (Shimadzu, Tokyo, Japan) coupled with a triple quadrupole, tandem mass spectrometer (GC-MSMS). The EO chemical composition was identified using a polar, capillary RTxi-5 Sil MS column (30.00 m long, 00.25 mm inside diameter, and a film thickness of 0.25). The composition of EO was presented as a proportion of the overall peak areas. EO phytochemicals were identified by comparing the resulted retention indices referring to those of the literature database [50].

### 3.5. Essential Oil Nanoemulsion Formation

Oil/water emulsion (O/W) was prepared at room temperature; the prepared Nanoemulsion consisted of 15 wt % *Lavandula angustifolia* essential oil/water and 5 wt % each surfactant (Span^®^ 80 and Tween^®^ 80). Nanoemulsions were prepared using the ultrasonic method [51]. The hydrophilic–lipophilic balance (HLB) method is a commonly employed approach for the selection of surfactants as emulsifying agents. HLB number of mixed surfactant system was calculated using the following equation:

(1) $$\mathrm{HBL}=\frac{(m_{A}\times {\mathrm{HLB}}_{A})+\left(m_{B}\times {\mathrm{HBL}}_{B}\right)}{m_{A}+m_{B}}$$

where m_A_ and m_B_ are the surfactants A and B mass, respectively. HLB_A_ and HLB_B_ are the HLB number of surfactants A and B, respectively. Tween^®^ 80 with a HLB value of 15.0 and Span^®^ 80 with a HLB value of 4.3, were selected for preparation of nanoemulsions. HLB value of 9 (Span^®^ 80, mA = 56% and Tween^®^ 80, mB = 44%) was defined based on Equation (1) with concentration of mixed surfactants in an emulsion system as determined by Shahavi et al. [51].

### 3.6. Synthesis of Gold Nanoparticles

*Lavandula angustifolia* essential oil was used as a reducing and stabilizing agent. Two (2) milliliters of *Lavandula angustifolia* essential oil were mixed with 30 mL of HAuCl_4_ solution (1 mmol/mL) followed by incubation at room temperature for 24 h. The reduction in Au^+3^ to Au^0^ was initially confirmed by visual inspection of color change from pale yellow to ruby red. The mixture was sonicated (Sonicator, Hielscher GmbH, Teltow, Germany) and centrifuged at 15,000 rpm for 11 min [52].

### 3.7. Synthesis of Gold–EO Conjugated Nanoparticles

The freshly synthesized AuNPs were added to the freshly prepared nanoemulsion (1:1) and the mixture was ultrasonicated (10 min) to allow for the conjugation between the gold nanoparticles and the nanoemulsions. The mixture was then incubated at room temperature for 24 h followed by centrifugation at 15,000 rpm for 11 min [53].

### 3.8. Characterization of the Prepared Nanosystems

The synthesized nanosystems were characterized using Fourier transform infrared spectroscopy (FTIR), transmission electron microscope and Zetasizer (Malvern Zetasizer Nano ZS, Malvern, UK) to determine the nanoparticles’ size and PDI according to Dorgham et al. [54].

### 3.9. Antibacterial and Antibiofilm Activity of the Prepared Nanosystems

The antibacterial activity was further evaluated via disc diffusion method, MIC, MBC and bacterial lethality curve [55]. Moreover, bacterial cultures (10^8^ CFU/mL) were grown overnight in liquid broth, then diluted (1:100) into fresh medium for minimal biofilm eradication concentration (MBEC) assay in accordance with Dorgham et al. [54].

### 3.10. Cytotoxic Effect

In the present study, WI-38 fibroblasts vero (normal) cells (ATCC: CCL-25) were used, containing 3000 cell/well (plated for 24 h). After incubation, 100 µL of different concentrations of extract in RPMI medium without fetal bovine serum was added, and then the plate was re-incubated for an additional 24 h in a CO_2_ incubator (37 °C, 5% CO_2_ and 90% relative humidity). After 24 h of incubation, 20 μL of MTT (3-(4,5-dimethylthiazol-2-yl)-2,5-diphenyltetrazolium bromide) solution was added/well and the plates were incubated for 3 h in a CO_2_ incubator (MTT reaction time). The formazan crystals (MTT byproduct) were re-suspended after centrifugation in 100 μL DMSO. Readings were measured at 570 nm. The % viability was calculated as follows:

(A_T_ − A_b_/A_C_ − A_b_) × 100 (2)

A_T_ = Mean absorbances of cells treated with different concentrations of each plant extract;
A_C_ = Mean absorbances of control untreated cells with culture medium only;
A_b_ = Mean absorbances of cells treated with vehicle of plant extract (RPMI media without fetal bovine serum).

The cytotoxicity assay of the compound was expressed as IC_50_ using the % viability calculated from the serial dilutions of each trial [56].

### 3.11. In Vitro Scratch Assay

Ten thousand (10,000) WI-38 cells/well (ATCC: CCL-25) were plated and incubated for 24 h in CO_2_ incubator (37 °C, 5% CO_2_, and 90% relative humidity). After incubation period, the culture medium was replaced with serum-free EMEM (Eagle’s Minimum Essential Medium) to wash the cell monolayer and scratched (wound mimicking) using a sterile 200 µL pipette tip. Afterward, 1.5 mL of complete medium or contained IC_50_ of the tested nanosystems was added and the plate was incubated again for another 24 h. The migrating cells in the denuded zone were observed and photographed using phase contrast microscopy. The relative wound size was quantitated by using the Image J version 1.49o software [57].

## 4. Conclusions

Wound healing is a complex process affected by many potential factors, which can delay healing. There is a high need for an effective treatment to protect the wound from contamination and promote such healing. The results of the present investigation revealed that *Lavandula angustifolia* essential oil possesses antibacterial and antifungal activities, in addition to the anti-inflammatory properties. A novel nanosystem of *Lavandula angustifolia* essential oil and gold nanoparticles was formulated, which promotes wound healing and combats bacterial infection. Dynamic light scattering (DLS) of the synthesized nanoparticles revealed that the nanoparticles’ size, polydispersity index and zeta potential of nano-*Lavandula angustifolia* were 199.5 nm, 0.404 and −13.6 mV; of nano-gold were 164.8 nm, 0.25 and +0.088 mV; and of nano-gold/nano-*Lavandula angustifolia* were 176.4 nm, 0.429 and −7.38 mV, respectively. In contrast, TEM micrographs revealed that the average diameter ranges of nano-*Lavandula angustifolia*, nano-gold, nano-*Lavandula angustifolia*/nano-gold were 96.8, 19.2 and 12.7 nm, respectively. The newly prepared nano-gold/nano-*Lavandula angustifolia* showed promising antibacterial activity as well as powerful wound healing efficacy. Moreover, the newly prepared nano-gold/nano-*Lavandula angustifolia* showed a cytotoxic effect against WI-38 fibroblasts vero (normal) cells (IC_50_ value = 0.089 mg/mL). Finally, the results showed that *Lavandula angustifolia* oil can be used as a candidate for speedy wound healing and bacterial contamination inhibition.

## Figures and Tables

**Table 1:** Antibacterial activity of the tested essential oil.

Tested Pathogens	*Lavandula angustifolia* EO		
	IZ (mm)	MIC (µg/mL)	MBC (µg/mL)
*P. mirabilis*	16.0 ± 1.0	64.0	256.0
*K. pneumoniae*	20.0 ± 2.0	32.0	128.0
*MRSA*	25.0 ± 2.0	16.0	128.0
*E. coli*	17.0 ± 5.0	64.0	256.0
*S. aureus*	28.0 ± 3.0	16.0	128.0
*A. baumannii*	17.0 ± 1.0	64.0	256.0

**Table 2:** GC–MS analysis of *Lavandula angustifolia* essential oil.

RT (min)	Area %	Compound
6.52	11.4	Cyclohexanol, 2-methyl-5-(1-methylethenyl)
8.26	60.2	Linalool
13.45	38.5	1,8-cineol

**Table 3:** Antibacterial and antibiofilm activity of the prepared nanosystems.

Nanosystems under Test	Measured Parameters	*P. mirabilis*
*Lavandula angustifolia* nanoemulsion	IZ (mm)	30.0 ± 2.0
	MIC (µg/mL)	128.0
	MBC (µg/mL)	512.0
	MBEC (µg/mL)	128.0
Gold nanoparticles	IZ (mm)	20.0 ± 1.0
	MIC (µg/mL)	256.0
	MBC (µg/mL)	512.0
	MBEC (µg/mL)	256.0
Nano-gold/nano-*Lavandula angustifolia*	IZ (mm)	45.0 ± 3.0
	MIC (µg/mL)	8.0
	MBC (µg/mL)	256.0
	MBEC (µg/mL)	16.0

**Table 4:** Wound healing activity of the prepared nanosystems.

Sample	Gap Width (µm)		Closure %
	0 h	48 h	
Control	124.03 ± 30.0	36.29 ± 6.0	70.74
Nano-*Lavandula angustifolia*	163.12 ± 12.0	19.65 ± 2.0	87.95
Nano-gold	155.63 ± 25.0	110.63 ± 2.0	28.91
Nano-*Lavandula angustifolia*/Nano-gold	164.12 ± 10.0	5.28 ± 0.5	96.78

## Data Availability

Not applicable.

## Supplementary Materials

The following supporting information can be downloaded at: https://www.mdpi.com/article/10.3390/molecules28196988/s1, Table S1: Microbial strains and their collection numbers.

## Sample Availability

Not available.
