# Supplementary material for: Nanoemulsion of Lavandula angustifolia Essential Oil/Gold Nanoparticles: Antibacterial Effect against Multidrug-Resistant Wound-Causing Bacteria
Source: Molecules. 2023 Oct 9;28(19):6988. doi: 10.3390/molecules28196988 (PMC10574385; doi:10.3390/molecules28196988)
Supplement: Supplementary file 1 [file molecules-28-06988-s001.zip › molecules-2635330-supplementary.pdf]

Table S1. Microbial strains and their collection numbers.

Microbial Strain	Collection Number
Acinetobacter baumannii	B370200
Proteus mirabilis	U98728
Staphylococcus aureus	B236844
Escherichia coli	U65814
Klebsiella pneumoniae	BA83700
